# NCOR1 modulates erythroid disorders caused by mutations of thyroid hormone receptor α1

**DOI:** 10.1038/s41598-017-18409-4

**Published:** 2017-12-22

**Authors:** Cho Rong Han, Sunmi Park, Sheue-yann Cheng

**Affiliations:** Laboratory of Molecular Biology, Center for Cancer Research, National Cancer Institute, National Institutes of Health, Bethesda, MD 20892 USA

## Abstract

Thyroid hormone receptor α (*THRA)* gene mutations, via dominant negative mode, cause erythroid abnormalities in patients. Using mice expressing a dominant negative TRα1 mutant (TRα1PV; *Thra1* ^*PV*/+^ mice), we showed that TRα1PV acted directly to suppress the expression of key erythroid genes, causing erythroid defects. The nuclear receptor corepressor 1 (NCOR1) was reported to mediate the dominant negative effects of mutated TRα1. However, how NCOR1 could regulate TRα1 mutants in erythroid defects *in vivo* is not known. In the present study, we crossed *Thra1* ^*PV*/+^ mice with mice expressing a mutant *Ncor1* allele (*NCOR1ΔID*; *Ncor1* ^*ΔID*^ mice). TRα1PV mutant cannot bind to NCOR1ΔID. The expression of NCOR1ΔID ameliorated abnormalities in the peripheral blood indices, and corrected the defective differentiation potential of progenitors in the erythroid lineage. The defective terminal erythropoiesis of lineage-negative bone marrow cells of *Thra1* ^*PV*/+^ mice was rescued by the expression of NCOR1ΔID. De-repression of key erythroid genes in *Thra1* ^*PV*/+^ *Ncor1* ^Δ*ID*/Δ*ID*^ mice led to partial rescue of terminal erythroid differentiation. These results indicate that the inability of TRα1PV to recruit NCOR1ΔID to form a repressor complex relieved the deleterious actions of TRα1 mutants *in vivo*. NCOR1 is a critical novel regulator underpining the pathogenesis of erythroid abnormalities caused by TRα1 mutants.

## Introduction

Thyroid hormone receptors (TRs) mediate the nuclear actions of thyroid hormone (T3) in growth, development, differentiation, and maintaining metabolic homeostasis. There are two TR genes, *THRA* and *THRB*, that encode three major T3 binding receptors, TRα1, TRβ1, and TRβ2. These receptors share high sequence homology in the DNA binding and T3 binding domains, but differ in the amino terminal A/B domain. The transcriptional activity of TR is dictated the type of thyroid hormone response elements on the promoter of T3 target genes, and is modulated by T3-dependent interaction with nuclear coregulatory proteins, e.g., nuclear corepressors and coactivators. The classical bimodal switch model of TR action is that in the absence of T3, TR complexed with the retinoid acid receptor (RXR) recruits corepressors to repress gene transcription. Binding of T3 releases corepressors to allow the liganded-TR-RXR to recruit coactivators to activate gene transcription. The best-studied nuclear corepressors are the nuclear receptor corepressor 1 (NCOR1) and silencing mediator of retinoid and thyroid hormone receptors (SMRT; NCOR2). These two corepressors share about 50% identity in the amino acid sequences, but have similar functional domains. However, the three receptor interaction domains located in the C-terminal region of these two corepressor proteins show some preferential avidity to associate with TRs. It is possible that these two corepressors could have non-overlapping functions to regulate TR actions in target tissues^1^.

Because TR isoforms share high sequence homology in the functional DNA and T3 binding domains, the question of whether TR isoforms have redundant or isoform-specific roles has been intensively studied. Mice deficient in *Thra*, *Thrb*, or both genes showed that TR isoforms have a redundant role as well as overlapping functions^2^. However, in mice expressing an identical knock-in dominant negative mutation (hereafter referred to as PV) in the *Thrb* or *Thra* genes, the phenotypic expression is distinct^3,4^. The *Thrb* ^*PV*^ mice exhibit the hallmark of resistance to thyroid hormone (RTHβ) with dysregulation of the pituitary-thyroid axis, hyperglycemia, and enlarged fatty livers^3,5,6^. In contrast, *Thra1* ^*PV*^ mice have nearly normal thyroid functions tests, but exhibit growth retardation, delayed bone development, and reduced fat mass and liver size^4,6,7^. These observations indicated that TR mutant isoforms exhibit distinct biological functions *in vivo* and predicted that mutations of TR subtypes could lead to different human diseases. While autosomal dominant resistance was first recognized in 1967^8^ and mutations of the *THRB* gene were identified to cause the disease (RTHβ) in 1989^9^, three patients with mutations of the *THRA* gene were not discovered until 2012^10,11^. Since then, 27 patients have been identified^10–13^. Indeed, similar to *Thra1* ^*PV*/+^ mice, in spite of nearly normal thyroid function tests, these patients exhibit classical hypothyroidism with growth retardation and delayed bone development, indicating resistance of target tissues to thyroid hormones (RTHα). The discovery of RTHα patients displaying symptoms distinct from those of RTHβ patients unambiguously shows that the *in vivo* molecular actions of TR mutant isoforms are distinct.

Interestingly, the mutated C-terminal sequences in TRα1PV share the identical truncated sequence in two RTHα patients^11^. Through use of *Thra1* ^*PV*/+^ mice, much has been learned about how mutated TRα1 led to bone abnormalities at the molecular levels^14,15^. Moreover, the *Thra1* ^*PV*/+^ mouse has been used as a preclinical model to test whether long-term treatment of T4 could be beneficial to patients with mutations of the *THRA* gene^16^. One notable pathological manifestation in patients with RTHα is erythroid disorders (e.g., anemia)^17^ that were not observed in RTHβ patients. Recently, we have shown that *Thra1* ^*PV*/+^ mice, similar to RTHα patients, also exhibited erythroid abnormalities^18^. We further elucidated that TRα1PV, via dominant negative action, impaired erythropoiesis by suppressing the expression of the key erythroid genes, the *Gata1*, *Klf1*, and their several downstream target genes in the bone marrow of *Thra1* ^*PV*/+^ mice^18^. These findings prompted us to further ascertain how the dominant negative actions TRα1PV is regulated in mediating the erythroid disorders *in vivo*. NCOR1 has been shown to modulate the *in vivo* dominant negative action of TRα1PV in the adipocytes^19^. Accordingly, we adopted the loss of function approach by crossing *Thra1* ^*PV*/+^ mice with mice expressing a mutant *Ncor1* allele (*NCOR1ΔID*; *Ncor1ΔID* mice) that cannot recruit TRα1PV mutant. Remarkably, we found that the disruption of the interaction of NCOR1 to complex with TRα1PV ameliorated the deleterious actions of TRα1PV on erythropoiesis. Thus, aberrant interaction of TRα1 mutants underpinning the pathogenesis of erythroid disorders. Importantly, the present studies uncovered NCOR1 as an important regulator in TRα1 signaling in erythropoiesis.

## Results

### Expression of NCOR1 ΔID reverts abnormal erythropoietic parameters and ameliorates defective progenitor differentiation capacity of *Thra1*^*PV*/+^ mice

**Figure 1:**
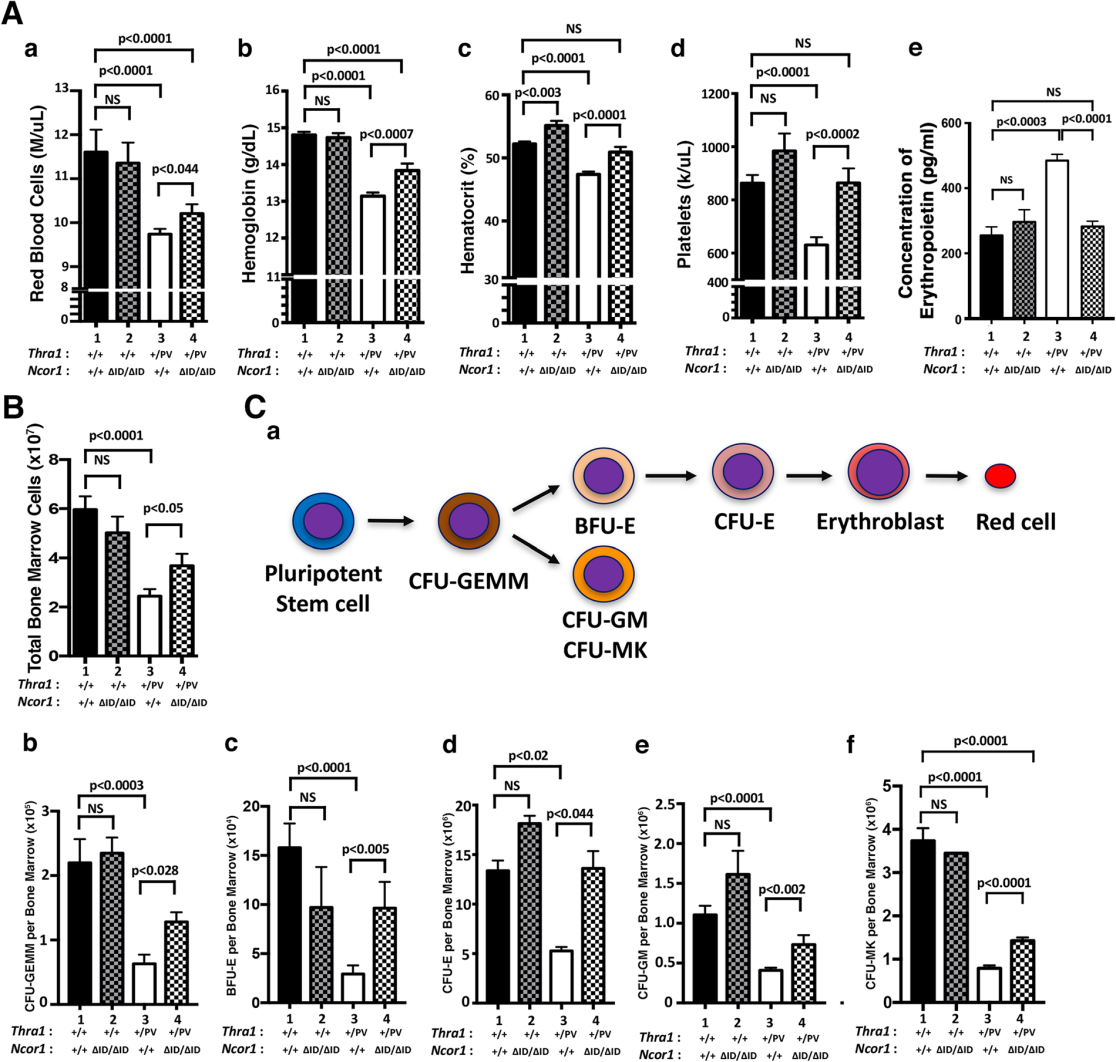
The expression of NCOR1ΔID partially corrects the abnormal blood indices, bone marrow cell number and colony forming ability of progenitors in *Thra1* ^*PV*/+^ mice. (**A** a–d) Peripheral blood indices as marked among adult mice (3–5 months old) with indicated genotypes (n = 13–58). P values are indicated. NS, not significant. (A-e) Serum EPO levels from mice with 4 genotypes as marked (n = 4–6) were determined as described in Methods, p values are indicated, NS, not significant. (**B**). Total bone marrow cells in among adult mice with indicated genotypes (n = 4–12). (**C**-a). Schematic representation of the erythroid lineage. The CFU-GEMM (panel b), BFU-E (panel c), CFU-E (panel d), CFU-GM (panel e) and CFU-MK (panel f) colonies from total bone marrow cells in mice with the indicated genotypes. The p values are indicated (duplicates in each assay; n = 5–7).

Previously, we have shown that peripheral erythropoietic indices were lower in *Thra1* ^*PV*/+^ mice than in wild-type (WT)^18^. Consistent with those findings, we found that the red blood indices were reduced 16.1% (red blood cell count), 11.2% (hemoglobin content), 9.2% (hematocrit) and 27% (platelets) as compared with WT mice (Fig. 1A, bars 3 versus bars 1 in panels, a,b,c and d). Remarkably, the expression of NCOR1ΔID in *Thra1* ^*PV*/+^ mice nearly completely corrected the decreased blood indices (bars 4 in all panels). These data indicated that the abnormal red blood cell indices of *Thra1* ^*PV*/+^ mice could be reverted by the expression of NCOR1ΔID.

It is known that anemia stress induces the expression of erythropoietin (EPO)^20,21^. Accordingly, we determined EPO levels in mice with four genotypes. *Thra1* ^*PV*/+^ mice which are anemic^18^, exhibited elevated EPO (bar 3, Fig. 1A-e). The reversal of anemic phenotypes by the expression of NCOR1ΔID in *Thra1* ^*PV*/+^ *Ncor1* ^Δ*ID*/Δ*ID*^ mice (bars 4 in Fig. 1A, panels a–d) led to the lowering of EPO (bar 4, Fig. 1A-e). These EPO data further support that the expression of NCOR1ΔID in *Thra1* ^*PV*/+^ *Ncor1* ^Δ*ID*/Δ*ID*^ mice ameliorated the erythroid disorders in *Thra1* ^*PV*/+^ mice.

Figure 1B shows that the expression of NCOR1ΔID partially corrected the decreased total bone marrow cells from a reduction of 58.0% in *Thra1* ^*PV*/+^ mice (bar 3 versus bar 1, Fig. 1B) to 38.2% in *Thra1* ^*PV*/+^ *Ncor1* ^*ΔID*/*ΔID*^ mice (bar 4 versus bar 1). There were no significant differences in the total bone marrow cells between WT mice and *Ncor1* ^*ΔID*/*ΔID*^ mice (bar 1 versus bar 2). That the expression of NCOR1ΔID could partially correct the deficiency in the total bone marrow cells of *Thra1* ^*PV*/+^ mice prompted us to ascertain the effect of the expression of NCOR1ΔID on the ability of colony forming units of the progenitors derived from colony forming unit (CFU) granulocyte-erythroid-monocyte-megakaryocyte (CFU-GEMM) downstream of hematopoietic stem cells (HSC; see Fig. 1C). The number of CFU-GEMM colonies was decreased 71.2% in *Thra1* ^*PV*/+^ mice compared with WT (Fig. 1C-b, bar 3), but was corrected to only 17% reduction in *Thra1* ^*PV*/+^ *Ncor1* ^*ΔID*/*ΔID*^ mice (Fig. 1C-b, bar 4). The number of burst-forming unit erythroid (BFU-E) and CFU erythroid (CFU-E) was also decreased 81.5% and 60.8%, respectively, in *Thra1* ^*PV*/+^ mice (bars 3 in panels c and d, Fig. 1C), but was corrected to the reduction of 59.4% and total recovery, respectively, in *Thra1* ^*PV*/+^ *Ncor1* ^*ΔID*/*ΔID*^ mice (bars 4 in panels c and d). The number of CFU-granulocyte (CFU-GM) and CFU-megakaryocyte (CFU-MK) was decreased 70.8% and 78.8%, respectively in *Thra1* ^*PV*/+^ mice (bars 3 in panels e and f, Fig. 1C), but was corrected to only reduction of 48.3% and 61.8%, respectively, in *Thra1* ^*PV*/+^ *Ncor1* ^*ΔID*/*ΔID*^ mice (bars 4 in panels e and f). These results indicated that the expression of NCOR1ΔID in *Thra1* ^*PV*/+^mice could ameliorate the impaired capacity of progenitor cells to differentiate from GEMM to the mature erythrocytes and megakaryocytes in *Thra1* ^*PV*/+^mice.

### Expression of NCOR1ΔID rescues the terminal erythropoiesis in Lin negative (Lin-) bone marrow cells

**Figure 2:**
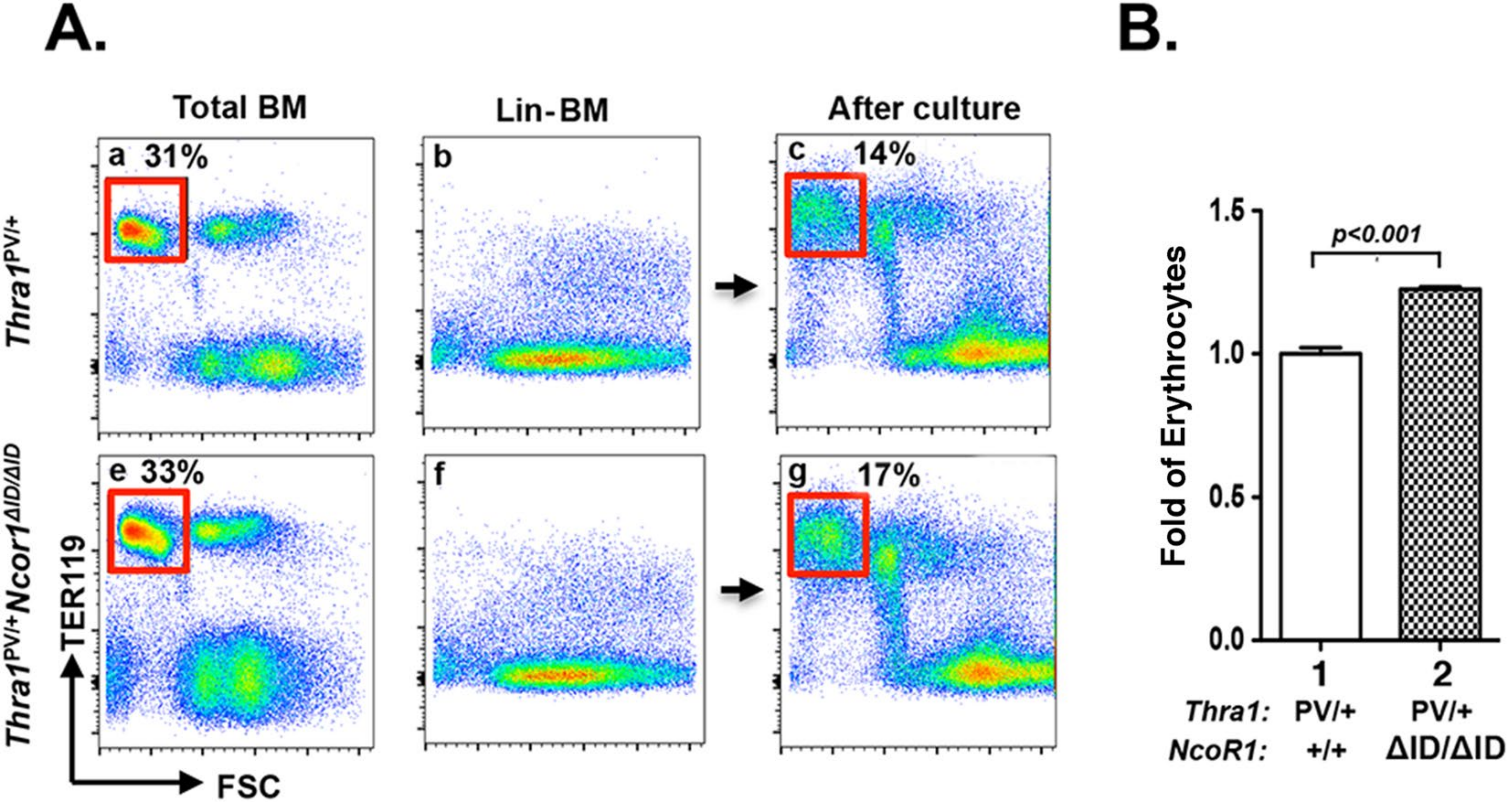
The expression of NCOR1ΔID significantly recovers the defective terminal erythroid differentiation in Lin-BM of *Thra1* ^*PV*/+^ mice. (**A**). Total bone marrow profile from *Thra1* ^*PV*/+^ mice (**A**-a) and *Thra1* ^*PV*/+^ *Ncor1* ^*ΔID*/*ΔID*^ mice (**A**-e). (Ter119+FSC^low^) population is boxed in red. Population of Lin-BM cells from *Thra1* ^*PV*/+^ (**A**-b) and *Thra1* ^*PV*/+^ *Ncor1* ^*ΔID*/*ΔID*^ (**A**-f) mice. Terminal induced differentiated Ter119 + FSC^low^ population is boxed in red (**A**-c for *Thra1* ^*PV*/+^ mice and A-g for *Thra1* ^*PV*/+^ *Ncor1* ^*ΔID*/*ΔID*^ mice). (**B**). Quantitative analysis shows the fold changes of erythrocytes after terminal erythroid differentiation of Lin-BM cells of *Thra1* ^*PV*/+^ mice and *Thra1* ^*PV*/+^ *Ncor1* ^*ΔID*/*ΔID*^ mice. P-values are indicated (mean ± SEM; n = 3).

Because patients with mutations of the *THRA* gene exhibit anemia, we focused our studies on the erythroid lineage. To further confirm that the effect of NCOR1ΔID on the maturation of erythrocytes in *Thra1* ^*PV*/+^mice, we used an *in vitro* terminal differentiation system^18^. Using an equal number of total bone marrow cells from *Thra1* ^*PV*/+^mice and *Thra1* ^*PV*/+^ *Ncor1* ^*ΔID*/*ΔID*^ mice (Fig. 2A-a and -e, respectively; the mature erythrocyte population shown in the gated boxes identified by Ter119+ with low FSC population), we isolated lineage depleted bone marrow cells (Lin-BM) as shown in Fig. 2A-b and -f, for *Thra1* ^*PV*/+^mice and *Thra1* ^*PV*/+^ *Ncor1* ^*ΔID*/*ΔID*^ mice, respectively. After induction of terminal differentiation, we found 14% and 17%, respectively, of Ter119+ with low FSC population (gated in red boxes). The quantitative comparison shows that the expression of NCOR1ΔID led to a 18% increase in matured erythrocytes in *Thra1* ^*PV*/+^ *Ncor1* ^*ΔID*/*ΔID*^ mice as compared with *Thra1* ^*PV*/+^mice (bar 2 versus bar 1, Fig. 2B). These findings indicated that the decreased number of mature erythrocytes in *Thra1* ^*PV*/+^ mice is markedly increased by the expression of NCOR1ΔID.

### TRα1PV-mediated repression of erythropoietic genes is de-repressed by the expression of NCOR1ΔID in the bone marrow of *Thra1*^*PV*/+^ mice

**Figure 3:**
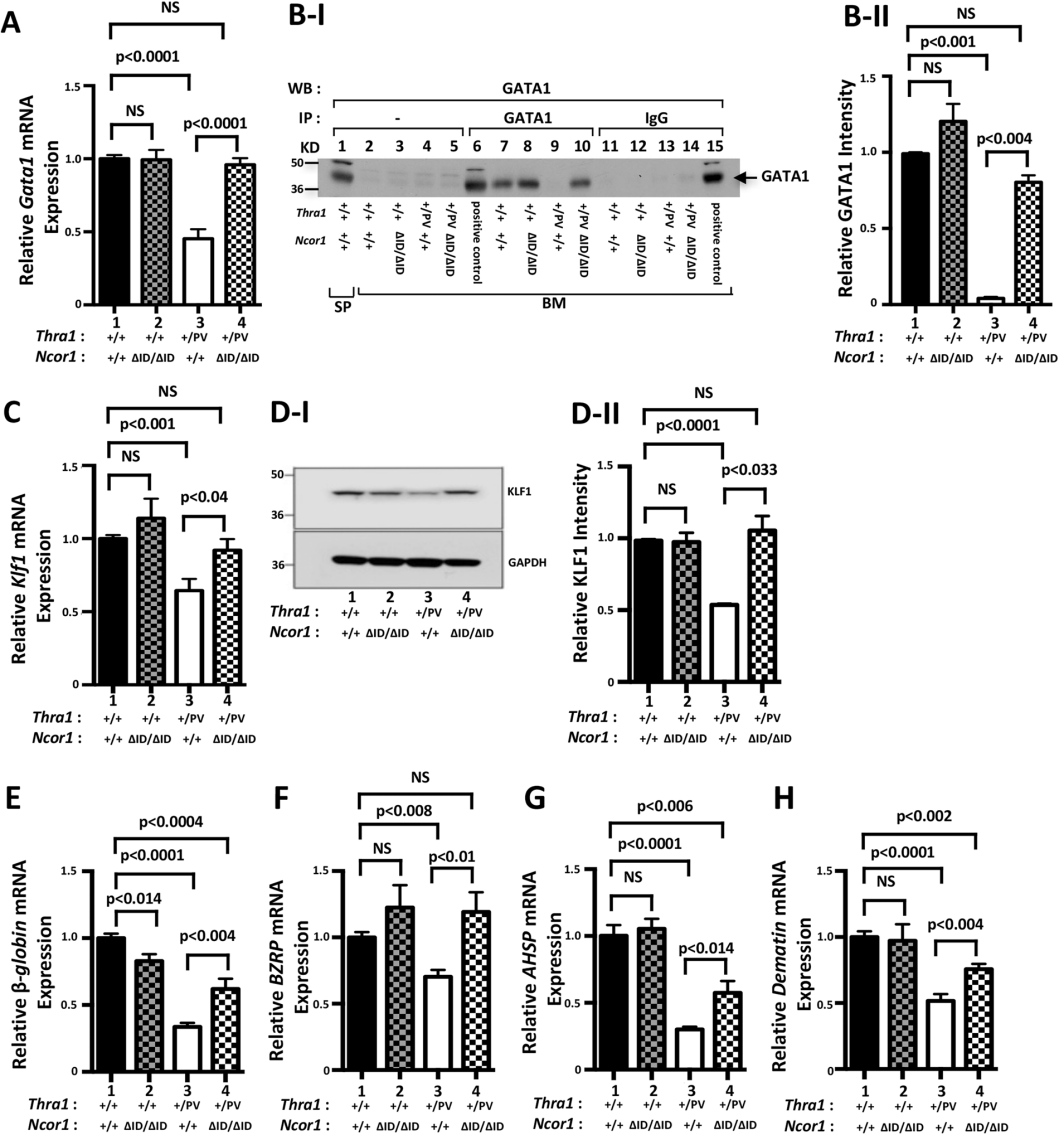
TRα1PV-mediated repression of key erythroid genes is de-repressed by the expression of NCOR1ΔID in the bone marrow of *Thra1* ^*PV*/+^ *Ncor1* ^Δ*ID*Δ*ID*^ mice. (**A**) Relative *Gata1* mRNA levels. (**B**) GATA1 protein levels determined by co-immunoprecipitation (**B**–I) (Full gel/blot is shown in the Supplemental Figure I) and quantitative data (**B**-II). (**C**) *Klf1* mRNA mice with indicated genotypes (P values indicated; mean ± SEM; n = 3 mice per group; each triplicates). (**D**–I) Western blot analysis of KLF1 protein abundance (Full gel/blot is shown in the Supplemental Figure II), and (**D**-II) quantification of the band intensities in (**D**-I) (n = 3 mice per group). Relative mRNA levels of *β-globin* (**E**), *Bzrp* (**F**), *Ahsp* (**G**), and *Dematin* (**H**) in total bone marrow cells of mice with indicated genotypes were determined by quantitative real-time PCR. Values are mean ± SEM (n = 2–3 mice per group; each triplicates).

To understand how the expression of NCOR1ΔID ameliorated the erythroid disorders in *Thra1* ^*PV*/+^ mice, we analyzed the expression of key erythroid regulators in *Thra1* ^*PV*/+^ *Ncor1* ^*ΔID*/*ΔID*^ mice. The GATA1 (erythroid transcription factor; GATA-binding factor 1) is essential for erythroid development by regulating a large ensemble of genes that mediate both the development and function of red blood cells^22,23^. We have recently shown that the *Gata1* gene is directly regulated by TRα1 and T3, and that TRα1PV acted to repress its expression in the bone marrow of *Thra1* ^*PV*/+^ mice^18^. Interestingly, the TRα1PV-mediated repression of the *Gata1* gene was totally de-repressed by the expression of NCOR1ΔID (bar 4 versus bar 3, Fig. 3A). The expression of *Gata1* mRNA in the bone marrow of *Ncor1* ^*ΔID*/*ΔID*^ mice was similar to that in WT mice (bar 2 in Fig. 3A). We further showed that GATA1 protein abundance was detected by co-immunoprecipitation assay in WT mice and *Ncor1* ^*ΔID*/*ΔID*^ mice (lanes 7 & 8, Fig. B-I), but was too low to be detected in the bone marrow of *Thra1* ^*PV*/+^ mice under identical experimental conditions (lane 9, Fig. 3B-I). Remarkably, a similar level of GATA1 protein as in WT mice was found in the bone marrow of *Thra1* ^*PV*/+^ *Ncor1* ^*ΔID*/*ΔID*^ mice (lane 10 versus lane 7, Fig. 3B-I; Also see the quantitative data shown in Figure B-II, bar 4 versus bar 1). These mRNA and protein data indicate that the expression of NCOR1ΔID led to reversal of TRα1PV-mediated repression of the *Gata1* gene.

We next evaluated the expression of the *Klf1* gene, which is a direct target gene of GATA1 and is involved in erythrocyte development^24^. The *Klf1* gene was also repressed by TRα1PV in the bone marrow of *Thra1* ^*PV*/+^ mice at the mRNA level (47% lower than in the WT mice; bar 3 of Fig. 3C), but was de-repressed to the level of WT mice by the expression of NCOR1ΔID in *Thra1* ^*PV*/+^ *Ncor1* ^*ΔID*/*ΔID*^ mice (bar 4 versus bar 1, Fig. 3C). Consistent with mRNA levels, we also found that the protein abundance of KLF1 was lower in the bone marrow of *Thra1* ^*PV*/+^ mice than in WT mice (lane 3 versus lane 1, Fig. 3D-I; quantitative data in bar 3, Fig. 3D-II), but was elevated to that of the WT level by the expression of NCOR1ΔID in *Thra1* ^*PV*/+^ *Ncor1* ^*ΔID*/*ΔID*^ mice (lane 4 versus lane 1, Fig. 3D-I; quantitative data in bar 4, Fig. 3D-II). The expression of *Klf1* mRNA (Fig. 3C, bar 2) and KLF1 proteins was not affected by NCOR1ΔID alone in *Ncor1* ^*ΔID*/*ΔID*^ mice (bar 2 in Fig. 3C and lane 2 in Fig. D-I and bar 2 in D-II).

We further analyzed the effects of NCOR1ΔID on the expression of other erythroid genes downstream of KLF1 in the bone marrow of mice with 4 genotypes.

In the bone marrow of *Thra1* ^*PV*/+^ mice, the expressions of the *β-globin*, peripheral type benzodiazepine receptor (*BZRP*), α-globin stabilizing protein (*AHSP*) and *dematin* mRNA were decreased by 66%, 30%, 70%, and 48%, respectively, (bar 3 versus bar 1 in Fig. 3E–H). The expression of NCOR1ΔID led to a total reversal in the expression of *Bzrp in Thra1* ^*PV*/+^ *Ncor1* ^*ΔID*/*ΔID*^ mice (bar 4 versus 3, Fig. 3F), and also partially de-repressed the expression of *β-globin, AHSP*, and *dematin* to lower reductions of 38%, 43%, and 24%, respectively, as compared with WT mice (bar 4 in Fig. 3E,G, and H, respectively). Except for the *β-globin* mRNA, whose expression was decreased (~17%) in *Ncor1* ^*ΔID*/*ΔID*^ mice as compared with wild type, the expression of NCOR1ΔID had no effect on the expression of *Bzrp*, *Ahsp*, and *dematin* in *Ncor1* ^*ΔID*/*ΔID*^ mice (bar 2 in Fig. 3F,G and H, respectively). Taken together, these results indicated that the repressed erythroid genes in *Thra1* ^*PV*/+^ mice were de-repressed by the expression of NCOR1ΔID in *Thra1* ^*PV*/+^mice.

### The inability of TRα1PV to interact with NCOR1ΔID leads to the reversal in the expression of the *Gata1* gene in *Thra1*^*PV*/+^***Ncor1***^***ΔID*/*ΔID***^ mice

**Figure 4:**
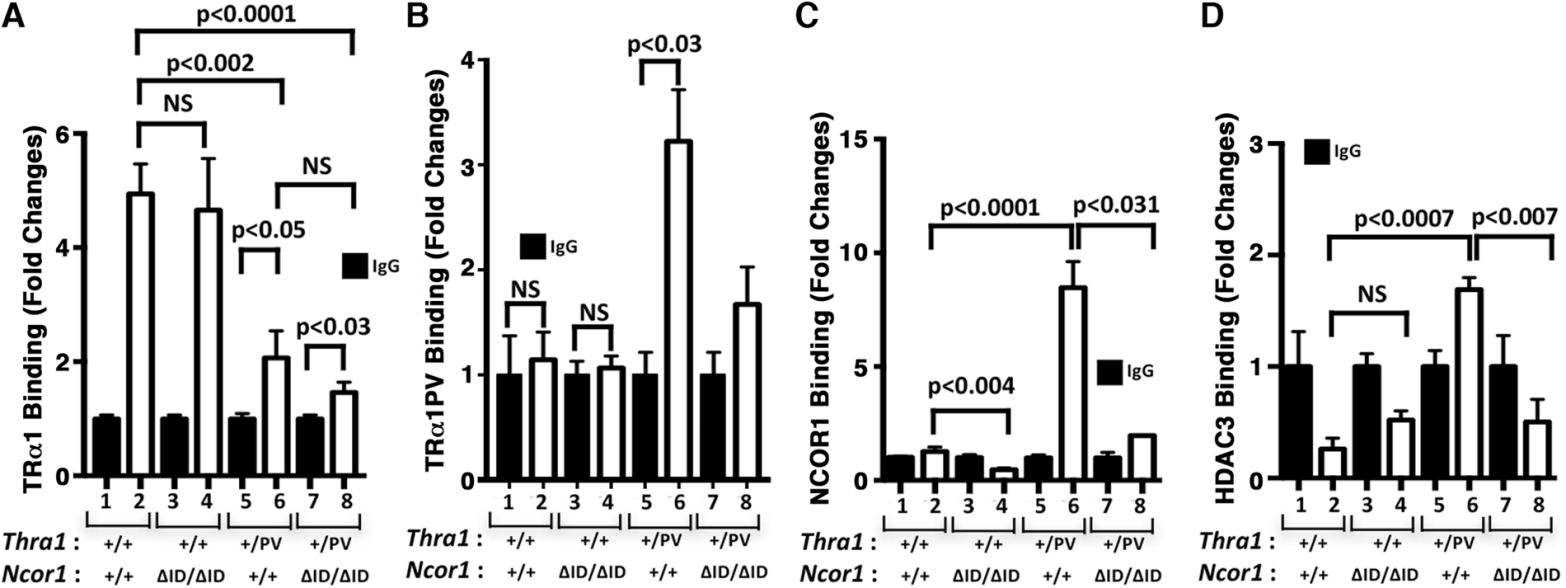
The inability of TRα1PV to interact with NCOR1ΔID leads to the reversal in the expression of the *Gata1* gene in *Thra1* ^*PV*/+^ *Ncor1* ^Δ*ID*/Δ*ID*^ mice. (**A**) ChIP assay was carried out using normal mouse IgG (solid bar) or anti-WT TRα1 (C4, n = 6–8) antibody (open bar), and (**B**), anti- TRα1PV-specific antibodies, T1, or normal rabbit IgG (solid bar) or (**C**) anti-NCOR1 antibody (open bar), (**D**) normal mouse IgG (solid bar) or anti-HDAC3 antibody (open bar), from total bone marrow cells of mice with indicated genotypes as described in Materials and Methods (n = 3–4 mice for each group).

Next we sought to understand the molecular basis by which the expression of NCOR1ΔID rescued the erythroid abnormalities caused by TRα1 mutants in *Thra1* ^*PV*/+^ mice. Previously, we have elucidated that the *Gata1* gene is directly and positively regulated by TRα1 via binding to one positive thyroid hormone response element (denoted as TRE2) on the promoter of the *Gata1* gene^18^. Using specific antibody against TRα1 (designated as C4) in ChIP analysis, we found a strong binding of TRα1 to TRE2 in the bone marrow of euthyroid WT mice (bar 2 versus 1, Fig. 4A). In mice expressing NCOR1ΔID, similar binding of TRα1 to TRE2 as in WT mice was found in the bone marrow of *Ncor1* ^*ΔID*/*ΔID*^ mice (bar 4 versus bar 2). As expected, a decreased binding of TRα1 to TRE2 was detected in the bone marrow of *Thra1* ^*PV*/+^ as well in *Thra1* ^*PV*/+^ *Ncor1* ^*ΔID*/*ΔID*^ mice (bars 6 and 8 versus bar 2, Fig. 4A) because anti-TRα1 antibody C4 cannot recognize TRα1PV. However, significant binding of TRα1 to TRE2 was detected (compare bars 6 to 5, Fig. 4A). To demonstrate the binding of TRα1PV to TRE2, we used anti-TRα1PV specific antibodies, T1, in the ChIP assays. As shown in Fig. 4B, T1 did not recognize TRα1 in the WT mice (bar 2, Fig. 4B), nor in *Ncor1* ^*ΔID*/*ΔID*^ mice (bar 4). In contrast, specific binding of TRα1PV to TRE2 was detected (compare bar 6 with bar 5), indicating that TRα1PV was bound to the promoter of the *Gata1* gene. A low but not significant binding of TRα1PV was detected in *Thra1* ^*PV*/+^ *Ncor1* ^*ΔID*/*ΔID*^ mice (bar 8, Fig. 4B). Using anti-NCOR1 antibody in ChIP analysis, we detected a significantly higher recruitment of NCOR1 by TRE2-bound TRα1PV to the promoter of the *Gata1* gene in *Thra1* ^*PV*/+^ mice (bar 6, Fig. 4C) than in *Thra1* ^*PV*/+^ *Ncor1* ^*ΔID*/*ΔID*^ mice (bar 8, Fig. 4C). Very low binding of NCOR1 to TRE2-bound TRα1 was detected in euthyroid WT mice. Virtually no NCOR1 binding to TRE2-bound TRα1 was detected in *Ncor1* ^*ΔID*/*ΔID*^ mice (bar 4 versus bar 2, Fig. 4C). NCOR1 is known to recruit histone deacetylase 3 (HDAC3) to form the repressor complex to suppress gene TR target gene transcription^25^. Using anti-HDAC3 antibody in ChIP analysis, we found that only TRE2 bound- TRα1PV-NCOR1 complex recruited HDAC3 to form repressor complex (bar 6, Fig. 4D). Taken together, these data supported the idea that the loss of interaction of TRα1PV with NCOR1ΔID led to reversal in expression of the *Gata1* gene in *Thra1* ^*PV*/+^ *Ncor1* ^*ΔID*/*ΔID*^ mice.

## Discussion

We have recently shown that the *Thra1* ^*PV*/+^ mouse faithfully recapitulates the erythroid disorders of patients with mutations of the *THRA* gene^18^. We further elucidated that the TRα1PV mutant acts to suppress the expression of several key erythroid genes in the bone marrow of *Thra1* ^*PV*/+^ mice, thereby causing erythroid defects. In the present studies, we aimed to understand the molecular mechanisms by which TRα1PV acted as a dominant negative suppressor to induce erythroid disorders. We took advantage of a mutant mouse that expresses the NCOR1ΔID mutant to test the hypothesis that the loss of the interaction of TRα1 mutants with NCOR1ΔID could reverse the deleterious effects of TRα1 mutants in erythropoiesis. Indeed, we found that the expression of NCOR1ΔID in *Thra1* ^*PV*/+^ mice led to partial reversal in the erythroid blood indices, corrected differential potential of progenitors in the erythroid lineage, increased the capacity of the terminal differentiation, and the reversal of the TRα1PV-mediated repression of key erythroid regulatory genes. These results indicated that aberrant association of NCOR1 with TRα1 mutants *in vivo* underlies the pathogenesis of erythroid disorders caused by TRα1 mutations.

The important role of NCOR1 in erythropoiesis has been documented in a mouse model deficient in NCOR1 (*Ncor1* ^−/−^ mice)^26^. *Ncor1* ^−/−^ mice exhibit anemia at E13.5, and the severity of the anemia increases with age, resulting in eventual death. Phenotypic analysis during embryo development showed that NCOR1 deficiency leads to defects in definitive erythropoiesis. The fetal liver size of *Ncor1* ^−/−^ mice was about half that of *Ncor1* ^-/+^ litter mates. Further, the BFU-E forming capacity was reduced in embryos at E13.5-E14.5 of *Ncor1* ^−/−^ mice. These observations clearly demonstrated that NCOR1 regulates erythroid development.

In line with these findings from *Ncor1* ^−/−^ mice, the present studies highlighted the critical regulatory role of NCOR1 in erythropoiesis using *Thra1* ^*PV*/+^ mice expressing NCOR1ΔID. Our studies focused on dissecting the erythroid defects in the bone marrow of adult mice. While the findings of these two studies were derived from two different mutant mice, the collective evidence allowed us to reach the same conclusion that NCOR1 plays critical roles in erythroid development. Moreover, the findings from these two studies are complementary in that the deficiency in functional NCOR1 caused defective erythropoiesis during development as shown in *Ncor1* ^−/−^ mice and that the defects could persist into adulthood as shown in *Thra1* ^*PV*/+^ *Ncor1* ^*ΔID*/*ΔID*^ mice. These two studies jointly indicate the need of NCOR1 in normal erythropoiesis not only during development, but also in the maintenance of normal erythropoiesis in adults. However, how NCOR1 was involved in regulating definitive erythropoiesis was not elucidated in *Ncor1* ^−/−^ fetal livers^26^.

By using *Thra1* ^*PV*/+^ *Ncor1* ^*ΔID*/*ΔID*^ mice, we found that one mechanism by which NCOR1 could impact erythropoiesis was via its aberrant association with TRα1PV. NCOR1 was recruited by TRE-bound TRα1PV on the promoter of the *Gata1* gene to suppress its expression, thereby impairing erythropoiesis^18^. On the basis of these findings, it is reasonable to postulate that TRα1 could be involved in the NCOR1 functions in definitive erythropoiesis during development. The association of NCOR1-TRα1 with certain erythroid regulatory genes to suppress their expression could be critical in definitive erythropoiesis. The loss of the suppression function of erythroid regulatory genes in *Ncor1* ^−/−^ mice would result in defective erythropoiesis during development. Identification of these genes in the future would help us understand the functions of not only TRα1, but also NCOR1 in erythropoiesis.

Previously we have shown that expression of NCOR1ΔID in *Thra1* ^*PV*/+^ mice ameliorated the abnormalities in the pituitary–thyroid axis and partially reverted infertility, growth retardation, impaired bone development, and lipid abnormalities^19^. The present studies showing that TRα1 mutants caused erythroid disorders further expanded the scope of the RTHα-resistant target tissues regulated by NCOR1 and further strengthened the conclusion that aberrant recruitment of NCOR1 by TRα1 mutants leads to clinical hypothyroidism in patients. However, it is noteworthy that the extent of the correction of abnormalities in the *Thra1* ^*PV*/+^ mice by the expression of NCOR1ΔID varies across target tissues. As shown previously, the mildly elevated serum total T3 and TSH levels were totally corrected to the basal levels of WT mice by the expression of NCOR1ΔID^19^. However, similar to those observed in growth, bone length, and white adipose tissues^19^, the correction in the erythroid defects was partial as shown in the incomplete recovery of total bone marrow cells and the colony forming capacity of progenitors in the erythroid lineage (see Fig. 1). The incomplete recovery would suggest that the dominant actions of TRα1 mutants could also be regulated by other nuclear corepressors such as NCOR2/SMRT. Erythropoiesis is a complex biological process and is modulated by large networks of regulators. NCOR1 could be affecting only a subset of erythropoietic genes. Still, the finding of partial corrections of the erythroid abnormalities by NCOR1 is a step forward in understanding how mutations of the THRA gene leads to erythroid defects in patients.

## Materials and Methods

### Mice and treatment

All animal studies were performed according to the approved protocols of the National Cancer Institute Animal Care and Use Committee. Mice harboring the mutated *Thra1* ^*PV*^ gene (*Thra1* ^*PV*^ mice) were prepared and genotyped by PCR as described earlier^4^. *Ncor1* ^*ΔID*^mice were prepared as described by^27^. The *Thra1* ^*PV*^ mice were crossed with *Ncor1* ^*ΔID*^mice to obtain different genotypes for studies. These mice were intercrossed several generations, and littermates with a similar genetic background were used in all experiments.

### Cells

Bone marrow cells were isolated from femurs and tibiae of mice with different genotypes (*Thra1* ^+/+^ *Ncor1* ^+/+^, *Thra1* ^+/+^ *Ncor1* ^*ΔID*/*ΔID*^, *Thra1* ^*PV*/+^ *Ncor1* ^+/+^ *, Thra1* ^*PV*/+^ *Ncor1* ^*ΔID*/*ΔID*^; age: 3–5 months). Single cell suspensions were prepared by passing bone marrow through a 70 µM cell strainer.

### Peripheral blood profile analysis

For analysis of complete blood counts, peripheral blood was collected in a heparinized microtube and analyzed by hematology analyzer (HEMAVET HV950FS, Drew Scientific, Miami Lakes, FL).

### Determination of serum mouse erythropoietin (EPO)

Erythropoietin levels were analyzed in mice (3–5 months old) with 4 genotypes. Collected blood were allowed to clot for 2 hours at room temperature before centrifuging for 20 minutes at 2000 X g. Serum EPO levels were measured by Quantikine mouse erythropoietin kit (Cat.no: MEP00B, R&D Systems, Minneapolis, MN, USA). Serum erythropoietin levels were quantified using a microplate reader set to 450 nm.

### Colony assays

Bone marrow cells were isolated from femurs and tibiae of mice with different genotypes at age of 3–5 months. To detect burst-forming units-erythroid (BFU-E), granulocyte/macrophage progenitor (CFU-GM), and multi-potential progenitor cells (CFU-GEMM) colonies, 4 × 10^4^ bone marrow cells were mixed with semisolid medium (Methocult GF M3434) by vortexing. The colony forming units-erythroid (CFU-E) colonies, 3.2 × 10^4^ bone marrow cells were mixed with semisolid medium (Methocult M3334). The colony forming units-megakaryocytes (CFU-Mk) colonies, 8 × 10^4^ bone marrow cells were mixed with semisolid medium (Methocult-c,04974, supplemented with 10 ng/mL Interleukin (IL)-3, 20 ng/mL Interleukin (IL)-6, 50 ng/mL thrombopoietin (TPO). All reagents and Methocults were purchased from STEMCELL Technologies, Vancouver, BC. Bone marrow cells in medium were seeded in duplicates on 6- well plates and cultured for 8 days for BFU-E, CFU-GM, and CFU-GEMM, 2 days for CFU-E, or 6 days for CFU-Mk for scoring.

### RNA extraction and quantitative RT-PCR

Total RNA was isolated from bone marrow cells using Trizol (Thermo Fisher Scientific, Waltham, MA). RT-qPCR was performed with one step SYBR Green RT-qPCR Master Mix (Qiagen, Valencia, CA). The mRNA level of each gene was normalized to the GAPDH (glyceraldehyde-3-phosphate dehydrogenase) mRNA level. The primer sequences used in RT-qPCR are listed in Supplemental Table 1.

###  Western blot analysis and co-immunoprecipitation

Cell lysates from bone marrow were prepared similarly as described^18^. The detection of KLF1 in the bone marrow of WT and mutant mice by western blot analysis was performed as described^18^. For the detection of GATA1 proteins in the bone marrow of WT and mutant mice, bone marrow lysates (600 μg each) were first immunoprecipitated with rat anti-GATA1 antibody (4 μg) or mouse IgG (4 μg; negative controls) followed by pulling down the enriched GATA1-anti-GATA1 antibody-complex with protein G-agarose beads. GATA1 proteins were subsequently detected by western blot analysis as described above using rabbit anti-GATA1 antibody. The antibodies used are listed in Supplemental Table 1.

### Chromatin immunoprecipitation assays (ChIP)

ChIP assay with bone marrow cells was performed as described previously^28^. Briefly, mouse bone marrow cells isolated from WT and mutant mice fixed in 1% of formaldehyde, washed, and sheared, followed by immunoprecipitation overnight at 4 °C with IgG (control), anti-TRα1 monoclonal antibody (C4), Anti-nuclear receptor corepressor 1 (NcoR1) antibody (ChIP grade; ab24552). Quantitative PCR was performed to detect the upstream fragment in *Gata1* genes with primer pairs (Supplemental Table 1). DNA binding was calculated as percentage of the input.

### *In vitro* terminal erythropoiesis assay

For lineage depleted bone marrow cell preparation, linage marker positive cells were depleted using the biotin based selection kit (cat# 19856, STEMCELL Technologies, Vancouver, BC) according to the manufacturer’s instructions. Lin-BM cells were seeded in fibronectin-coated wells (Corning Inc, Corning, NY). To induce erythropoiesis, Lin- BM cells were cultured as described^29^. On the first day, Lin- BM cells were cultured in Iscove’s Modified Dulbecco’s Medium (IMDM medium) supplemented with 15% FBS, 1% detoxified bovine serum albumin (BSA; Sigma-Aldrich, St. Louis, MO), 200 µg/mL holo-transferrin (Sigma-Aldrich, St. Louis, MO), 10 µg/mL recombinant human insulin (Sigma-Aldrich, St. Louis, MO), 2 mM L-glutamine, 10^−4^ M β-mercaptoethanol, 50 units/ml penicillin G, 50 µg/ml streptomycin (Thermo Fisher Scientific, Waltham, MA) and 2 U/mL Epo (STEMCELL Technologies, Vancouver, BC). The medium was replaced with IMDM with 20% FBS, 2 mM L-glutamine and 10^−4^ M β-mercaptoethanol for erythroid differentiation for the second and third day.

### Flow cytometry analysis

All antibodies used in flow cytometry were from eBiosciences (Thermo Fisher Scientific, Waltham, MA). To exclude dead cells from analysis, 7-aminoactinomycin D (7-AAD) was used and doublets were excluded using forward and side scatter width parameters. All cells for FACS analysis were immune-stained at 4 °C in PBS/5% FBS/1 mM EDTA buffer. For the erythrocytes, bone marrow cells were analyzed without lysis of red blood cells. Antibodies used to determine terminal erythropoiesis using Lin- bone marrow cells are as follows: anti-Ter119 (TER-119, APC-eFluor® 780) and anti-CD44 (IM7, eFluor® 450). The flow cytometry analyses were performed on a BD LSR II flow cytometer (BD Bioscience, San Jose, CA) and analyzed with FloJo, LLC (Tree Star Inc, Ashland, OR).

### Statistical analysis

All statistical analyses and the graphs were performed and generated using GraphPad Prism version 6.0 (GraphPad Software, La Jolla, CA). Student’s *t* test was used to examine whether differences between groups are statistically different from each other. *P* < 0.05 is considered statistically significant. All data are expressed as mean ± SEM.

## Electronic supplementary material

Tables and figures
