# Supplementary material for: NCOR1 modulates erythroid disorders caused by mutations of thyroid hormone receptor α1
Source: Sci Rep. 2017 Dec 22;7:18080. doi: 10.1038/s41598-017-18409-4 (PMC5741760; doi:10.1038/s41598-017-18409-4)
Supplement: Supplementary file 1 — Tables and figures [file 41598_2017_18409_MOESM1_ESM.pdf]

NCOR1 modulates erythroid disorders caused by mutations of thyroid hormone receptor α1

Cho Rong Han, Sunmi Park, and Sheue-yann Cheng*

Laboratory of Molecular Biology, Center for Cancer Research, National Cancer Institute, National Institutes of Health, Bethesda, MD, USA 20892

*Correspondence should be addressed to Dr. Sheue-yann Cheng, Laboratory of Molecular Biology, National Cancer Institute, 37 Convent Drive, Room 5128, Bethesda, MD 20892-4264, Tel: (240) 760-7828; Fax: (240)-541-4498

E-mail: *chengs@mail.nih.gov*

Table 1. Primer List

1. RT-qPCR Primers

	Forward primer	Reverse primer
gapdh	5'-CGTCCCGTAGACAAAATGGT-3'	5'-AATTTGCCGTGAGTGGAGTC-3'
gata1	5'-ATCAGCACTGGCCTACTACAGAG-3'	5'- GAGAGAAGAAAGGACTGGGAAAG-3'
klf1	5'-TCTGAGGAGACGCAGGATTT-3'	5'-CTCGGAACCTGGAAAGTTTG-3'
β -globin	5'-GGCAGGCTGCTGGTTGTCTA-3'	5'-GCCATGGGCCTTCACTTTG-3'
bzrp	5'-GCAGATGGGCTGGGCCTT-3'	5'-AGGCCAGGTAAGGGTACAGCAA-3'
ahsp	5'-CTCAGCACCATTAGACTTGAA-3'	5'-TGCTGATCCAGCAGAACATTAAACTC-3'
dematin	5'- ACCGCATGAGGCTTGAGAGG-3'	5'-TCTTCTTAAGTTCGTTCCGCTTCC-3'

2. Chip-qPCR primers

	Forward primer	Reverse primer
gata1	5'- GGTCTCAAATGGAAGCCTGA -3'	5' – CGGCAGAAATTGTGCATCT -3'

3. Antibodies

	Method	Source	Dilution	Company	Catalog
**Anti-GATA1antibody**	Immuprecipitation (IP)	Rat	4 ug	Santa Crus Biotechnology	#SC-265
**Anti-GATA1antibody**	Western blot (WB)	Rabbit	1:1000	Abcam	#ab28839
**Anti-KLF1 antibody**	Western blot (WB)	Goat	1:200	Santa Crus Biotechnology	#SC-27194
**Anti-C4 antibody**	Chromatin Immunoprecipitation (ChIP)	Mouse	5 ug	NIH/NCI	-
**Anti-NCOR1 antibody**	Chromatin Immunoprecipitation (ChIP)	Rabbit	5 ug	Abcam	#ab24552
**Anti-HDAC3 antibody**	Chromatin Immunoprecipitation (ChIP)	Rabbit	5 ug	Santa Crus Biotechnology	#SC-11417X

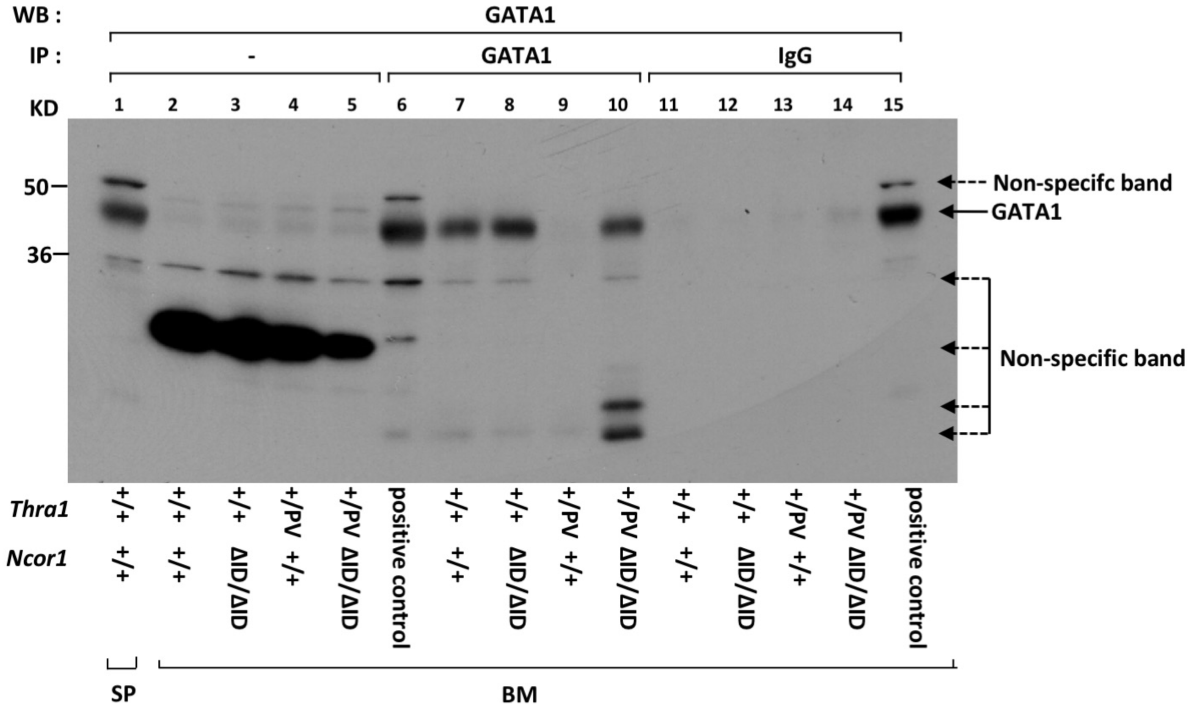

Supplemental Figure I. This figure displays the full-length gel/blot for Figure 3 (B-I) shown in the text/Results. The identification of GATA1 bands was based on the expected molecular weight shown in the positive control (lane 15).

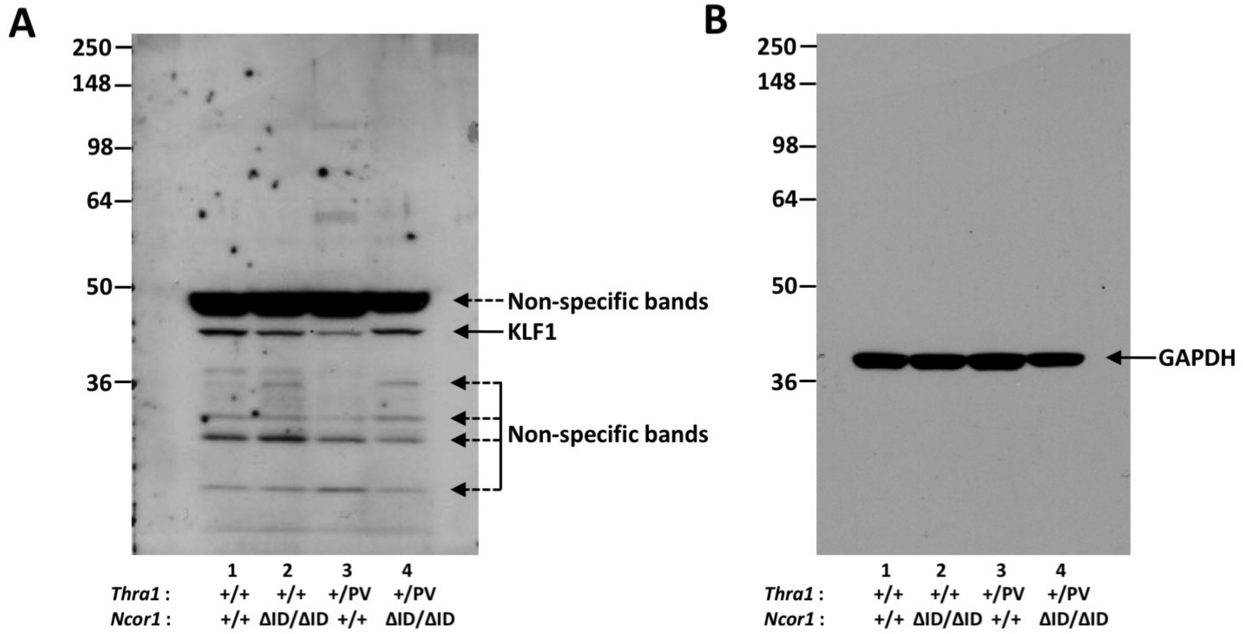

Supplemental Figure II. This figure displays the full-length gel/blot for Figure 3 (D-I) shown in the text/Results. The identification of KLF1 bands was based on the expected molecular weight, which was also shown in other publications. Panels A and B are from the same gel/blot. Panel B was re-blotted from the same blot using anti-GAPDH antibody for loading control.
